# Clinical Characteristics and Management of Anemia in Nonagenarians: A Retrospective Cohort Study

**DOI:** 10.7759/cureus.100583

**Published:** 2026-01-01

**Authors:** Kosuke Obama, Atsushi Kuroda, Atsushi Kusumoto, Naruhito Wakimaru, Hiroji Yanamoto, Manabu Setoguchi, Kenichi Kodera, Kentaro Hokonohara

**Affiliations:** 1 Internal Medicine, Takusyokai Memorial Hospital, Satsumasendai, JPN; 2 Neurological Surgery, Takusyokai Memorial Hospital, Satsumasendai, JPN; 3 Surgery, Takusyokai Memorial Hospital, Satsumasendai, JPN

**Keywords:** aging, anemia, egfr, erythropoietin, older patients

## Abstract

Introduction

The global increase in the older adult population has been accompanied by a rising prevalence of anemia, which is a condition linked to various negative health outcomes. This study assessed anemia in hospitalized patients aged 90 and older, aiming to assess its age-related characteristics.

Methods

This retrospective cohort study was conducted at a chronic-phase care hospital, focusing on patients aged 90 years or older who were hospitalized in November 2024. Patients with active cancer, autoimmune diseases, hematological disorders, or iron deficiency anemia were excluded. Data related to age, sex, hemoglobin, estimated glomerular filtration rate (eGFR), and albumin levels were extracted from routine blood test results of the target patient population. Based on these results, a statistical analysis of the factors affecting anemia was conducted.

Results

Pearson’s correlation analyses revealed that age and eGFR were not significantly correlated with hemoglobin. In contrast, serum albumin exhibited a significant positive correlation with serum hemoglobin (r = 0.329, 95%CI: 0.116 to 0.513, p = 0.003). In the multiple linear regression analysis, serum albumin remained a significant independent predictor of serum hemoglobin after adjusting for other variables (β = 1.3, 95%CI 0.56 to 2.1, p < 0.001). Our results also indicated that many cases exhibited no evidence of anemia despite a significant reduction in eGFR. Conversely, anemic cases presumably attributable to decreased erythropoietin production were observed even among patients with high eGFR values.

Conclusions

Anemia in the older adult population is primarily related to inflammation and malnutrition, with additional contributions from age-related changes in individual organs such as the kidneys and bone marrow. In the management of geriatric anemia, it is essential to consider organ-specific aging processes associated with underlying inflammatory conditions, with particular attention to impaired erythropoietin production that occurs independently of eGFR decline.

## Introduction

In recent years, the growing global population of older adults has led to a rise in the prevalence of anemia within this demographic. According to the World Health Organization criteria, anemia affects up to 40% of nursing home residents and hospitalized older adults [1]. This trend has prompted extensive research on the clinical significance of anemia in older populations. Anemia in older adults is associated with cognitive decline [2,3], insomnia [4], depression [5], reduced quality of life [6,7], and the onset of cardiovascular disease [8]. However, the etiology of anemia is multifactorial, involving numerous contributors such as chronic inflammation, malnutrition, decline in renal function with reduced erythropoietin production, and impaired bone marrow function [9]. In addition, a subset of cases is classified as unexplained anemia [10]. Although the contributing factors of anemia may become increasingly complex with advancing age, the analysis of anemia pathophysiology in the very elderly individuals may, conversely, provide valuable insights into the biological impact of aging. Therefore, the present study examined anemia in nonagenarians (patients aged 90 years and older), aiming to investigate the effects of aging on anemia.

## Materials and methods

This retrospective cohort study on anemia in older patients was conducted at the Takusyokai Memorial Hospital, Satsumasendai, Japan. This hospital provides long-term care with a focus on rehabilitation for patients in the chronic phase with stable medical conditions. Participants were individuals aged 90 years and older who were hospitalized at our institution in November 2024. The study was approved by the Institutional Ethics Committee of the Takusyokai Memorial Hospital (No. 06-04, dated March 6, 2025).

Study population

Patients with active malignancies, autoimmune diseases, hematological disorders, or iron deficiency anemia were excluded. Age, sex, hemoglobin (Hb), estimated glomerular filtration rate (eGFR), and albumin values were extracted from routine blood test results of the target patient population. Based on these results, we conducted a statistical analysis of the factors affecting anemia. Moreover, to investigate the underlying causes of anemia in selected individual cases, detailed analyses were conducted. Chemistry parameters were measured using standard automated techniques. Serum erythropoietin (EPO) concentration was measured using a chemiluminescent enzyme immunoassay with a normal range of 4.2-23.7 IU/L. Although the widely recognized WHO criteria exist for defining anemia, a substantial proportion of the very elderly population would be classified as anemic, particularly those with mild anemia. From the perspective of considering the pathophysiology of anemia and indications for therapeutic intervention, the present study defined anemia as hemoglobin levels below 11 g/dL, based on several previous reports.

Statistical analyses

Factors associated with serum hemoglobin were examined using Pearson’s correlation coefficients and simple and multiple linear regression analyses. For the correlation analyses, only continuous variables (age, serum albumin, and eGFR) were included, whereas sex was not analyzed because it is categorical. For the regression analyses, explanatory variables included age (years), sex (male/female), serum albumin (g/dL), and eGFR (mL/minute/1.73 m²). Each variable was individually incorporated into the simple linear regression models, whereas in the multiple linear regression model, all four variables were simultaneously included. Regression coefficients (β) and correlation coefficients (r) with 95% confidence intervals (95% CI) and p-values were calculated. A p-value of <0.05 was considered statistically significant. Statistical analyses were performed using R software (version 4.5.1; R Foundation for Statistical Computing, Vienna, Austria).

## Results

**Figure 1:**
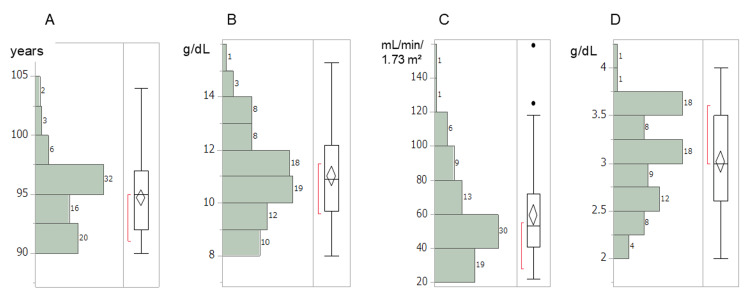
Histogram distribution of (A) age, (B) hemoglobin, (C) estimated glomerular filtration rate (eGFR), and (D) albumin in the 79 patients studied.

A total of 79 patients (67 women and 12 men) were included in this study. Reflecting the characteristics of our institution, many patients exhibited poor performance status (PS) and were presented with comorbidities such as cardiovascular disease, sequelae of cerebrovascular disorders, and dementia. The collected data were derived from routine laboratory examinations, and no missing data were identified. The severity of anemia was generally mild, with all patients having Hb levels above 8 g/dL. Hb levels below 11 g/dL were observed in 41 patients (51.9%). None of the patients with anemia exhibited leukopenia (< 3.00 × 10⁹/L) or thrombocytopenia (< 100× 109/L). Approximately half of the cases also demonstrated decreased albumin levels and eGFR (Figure 1). Given the advanced age and poor PS of the patients, invasive diagnostic procedures, including bone marrow examinations, were not performed. 

**Figure 2:**
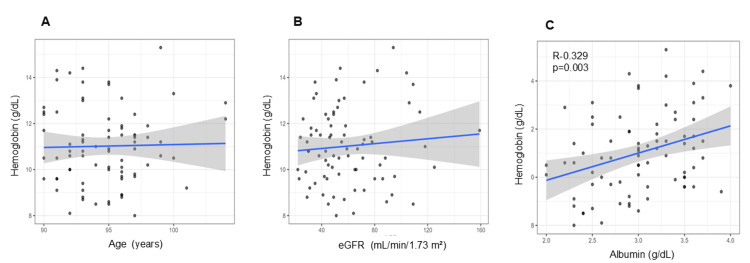
Correlations between hemoglobin levels and age, estimated glomerular filtration rate (eGFR), and albumin. In Pearson’s correlation analyses, (A) age (r = 0.023, 95%CI: –0.199 to 0.243, p = 0.839) and (B) eGFR (r = 0.086, 95%CI: –0.137 to 0.302, p = 0.449) were not significantly correlated with hemoglobin. (C) Serum albumin showed a significant positive correlation with serum hemoglobin (r = 0.329, 95%CI: 0.116 to 0.513, p = 0.003).

In Pearson’s correlation analyses, age (r = 0.023, 95%CI: -0.199 to 0.243, p = 0.839) and eGFR (r = 0.086, 95%CI: -0.137 to 0.302, p = 0.449) were not significantly correlated with Hb (Figure 2A, 2B). In contrast, serum albumin exhibited a significant positive correlation with serum Hb (r = 0.329, 95%CI: 0.116 to 0.513, p = 0.003) (Figure 2C). In the multiple regression analysis, serum albumin remained a significant independent predictor of serum hemoglobin after adjusting for other variables (β = 1.3, 95%CI: 0.56 to 2.1, p < 0.001). Age, sex, and eGFR demonstrated no statistically significant associations with serum Hb (Table 1).

**Table 1:** Simple and multiple linear regression analysis of factors affecting hemoglobin levels eGFR, estimated glomerular filtration rate

Parameters		Simple linear regression			Multiple linear regression		
		β	95% CI	p-value	β	95% CI	p-value
Age		0.01	-0.11, 0.14	0.839	0.04	-0.07, 0.16	0.446
Sex	Male	-	-	-	-	-	-
	Female	-0.81	-1.8, 0.22	0.121	-0.84	-1.9, 0.18	0.104
Albumin		1.1	0.39, 1.9	0.003	1.3	0.56, 2.1	<0.001
eGFR		0.01	-0.01, 0.02	0.449	0.01	-0.001, 0.002	0.273

**Figure 3:**
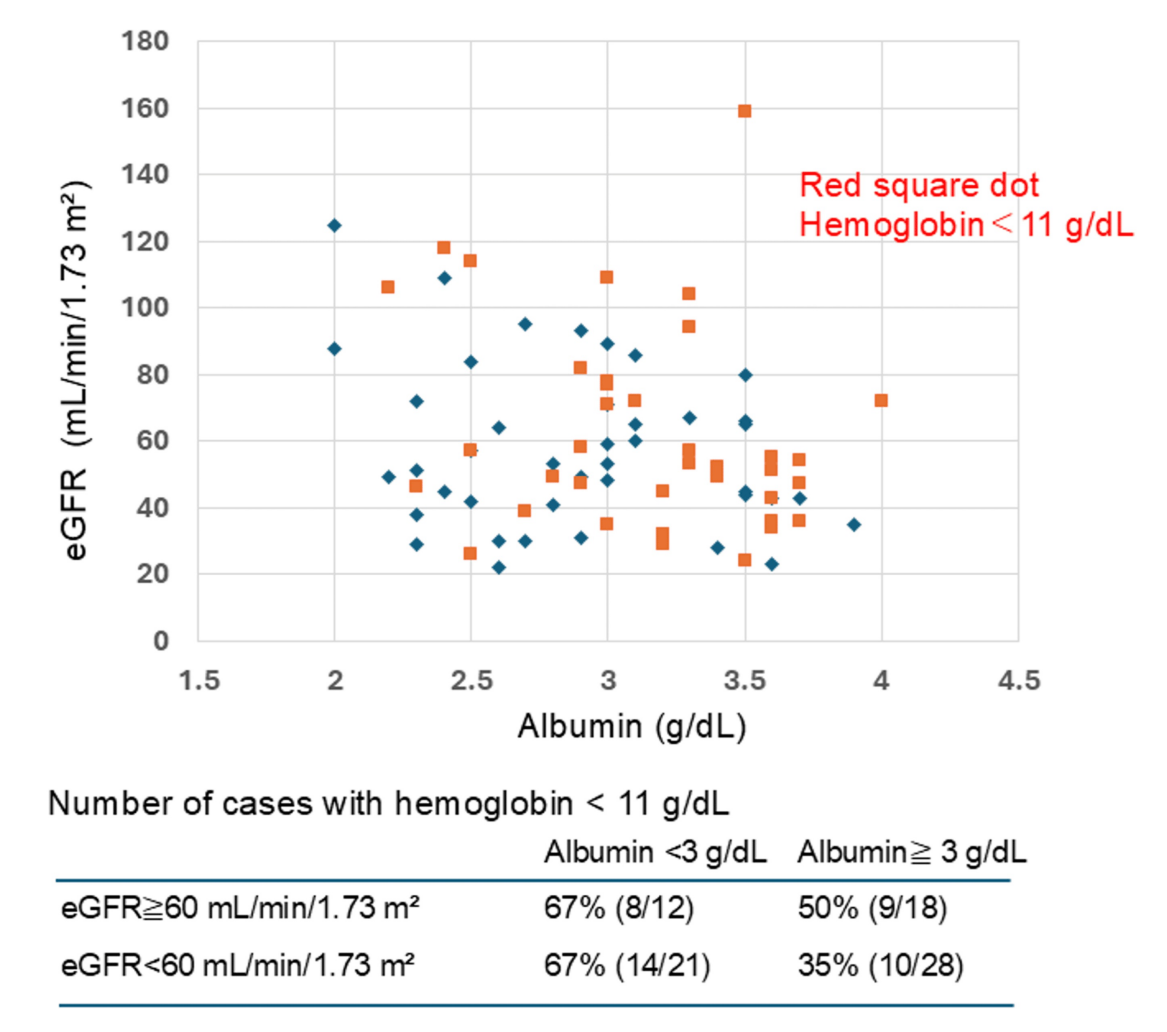
A scatter plot was generated with estimated glomerular filtration rate (eGFR) and albumin as the two axes. Cases with anemia are distributed across a wide range on both axes.

To evaluate the status of individual cases that could not be confirmed through statistical analysis, a scatter plot was generated with eGFR and albumin as the two axes (Figure 3, red dots indicate cases with Hb <11 g/dl). This plot demonstrated that 51% of cases with eGFR <60 (25 of 49 cases) did not exhibit anemia (Hb <11 g/dl). Conversely, among cases with eGFR ≥60 mL/min/1.73 m² and albumin levels ≥3 g/dl, 50% (nine of 18 cases) presented with anemia. Further investigation into the etiology of anemia in six of these nine cases suggested that impaired erythropoietin (EPO) production capacity, which was discordant with eGFR levels, may have been a contributing factor (Table 2).

**Table 2:** Detailed analysis of cases with eGFR ≥60 mL/minute/1.73m² and albumin levels ≥3 g/dL All cases were female. Hb, hemoglobin; eGFR, estimated glomerular filtration rate; Alb, albumin; Ret, reticulocyte; CRP, C-reactive protein; EPO, erythropoietin; UIBC, unsaturated iron binding capacity; B12, vitamin B12; Folic, folic acid; IgG, immunoglobulin G; TSH, thyroid stimulating hormone.

S. No.	Age (years)	Hb (g/dL)	eGFR (mL/min/1.73m²)	Alb (g/dL)	Ret (%)	CRP (mg/dl)	EPO (mlU/ml)	Fe (µg/dL)	UIBC (µg/dL)	Ferritin (ng/ml)	B12 (pg/ml)	Folic (ng/ml)	IgG (mg/dL)	TSH (µlU/ml)
1	91	9.7	67	3.6	2.1	0.3	11.9	30	283	37.8	1258	60	1593	2.8
2	91	10.8	165	3.3	2.8	0.85	24.6	74	165	566	2240	17.4	1583	0.2
3	95	8.6	89	3.3	2.4	0.3	27.5	38	169	260	276	5.5	1655	2.3
4	96	9.7	88	3.1	1.7	0.23	16.1	63	124	128	325	5.1	2230	1.65
5	96	9.6	86	3.1	1.8	0.47	10.1	41	293	13.7	825	14.9	1317	4.2
6	94	9	70	3.1	1.8	1.7	11.9	31	254	25.9	1200	20	2609	0.97

## Discussion

The present study investigated the current relationship between anemia and aging by focusing on hospitalized patients aged 90 years and older. As patient backgrounds become increasingly complex with age, this older population has rarely been the subject of prior research, making our findings valuable for understanding the present landscape of aging and anemia. Our study revealed a significant correlation between decreased albumin and Hb levels, suggesting that chronic inflammation or malnutrition contributes to anemia in older adults. Inflammation induces reduced hepatic albumin synthesis, increased hepcidin production (which impairs iron utilization), suppressed EPO production in the kidneys [11], and diminished bone marrow responsiveness to EPO. This cascade is considered a common underlying mechanism in age-related (senescent) anemia. However, given the limited statistical significance of this correlation, further investigation into the multifactorial causes of anemia in this elderly demographic is warranted.

We observed no significant correlation between eGFR and Hb levels. Contrary to commonly observed findings, a substantial number of cases with markedly reduced eGFR did not have anemia in our present study. Further investigation of anemic cases with preserved eGFR and albumin levels suggested that many cases developed anemia due to decreased EPO production capacity even when the eGFR was maintained. These findings are consistent with those of a previous study demonstrating the absence of elevated EPO levels in older patients with anemia [12]. Although the available information in the present analysis is limited, our results indicate that in older patients, glomerular filtration capacity and erythropoietin production capacity may undergo divergent age-related decline. Additionally, a concomitant decrease in bone marrow responsiveness to EPO can be reasonably anticipated.

**Figure 4:**
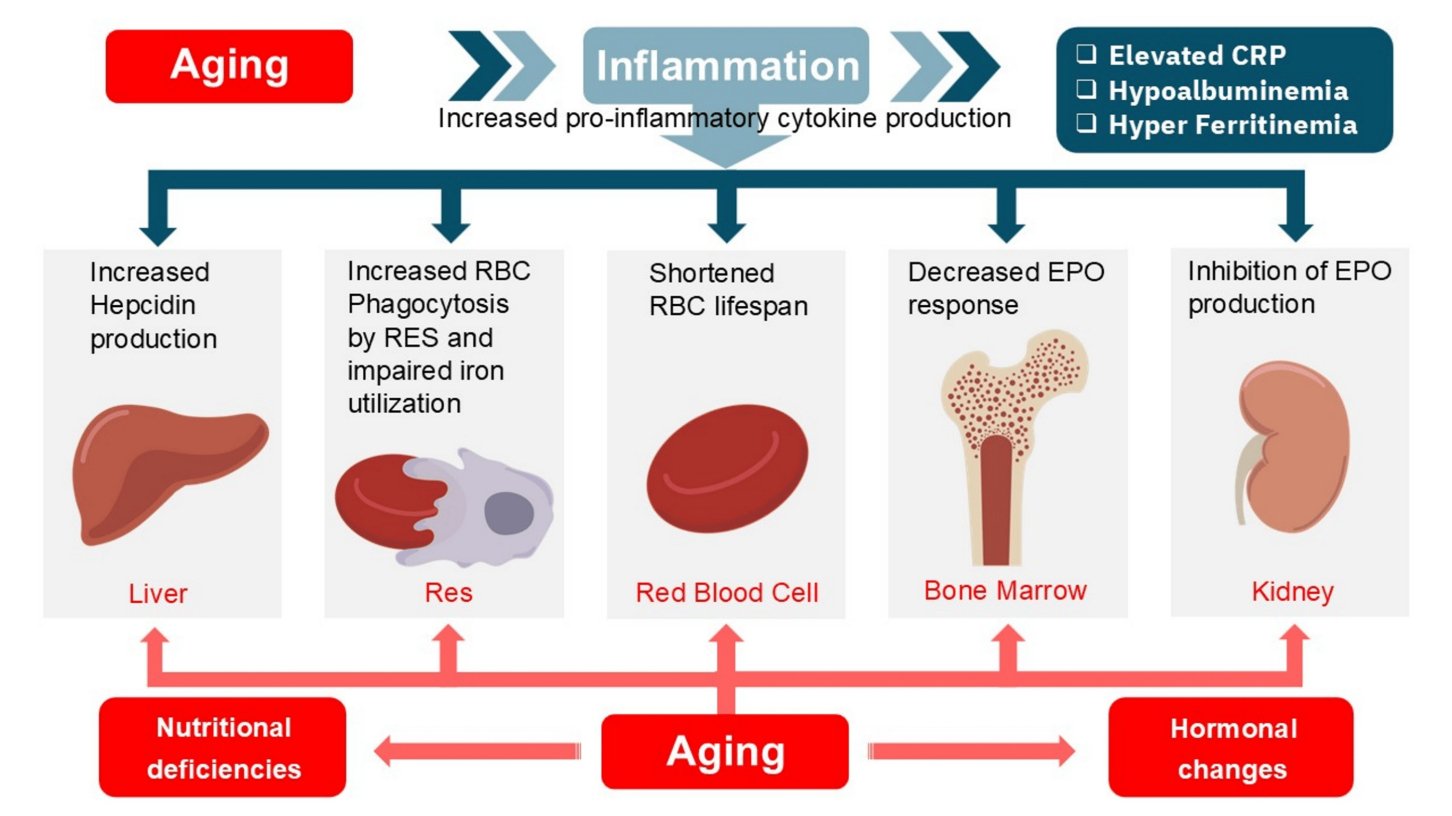
Pathophysiology of anemia in nonagenarians Inflammatory conditions induce anemia through various mechanisms in a substantial proportion of elderly individuals. Additionally, age-related functional decline of specific organs contributes to the pathophysiology of anemia in this population. In cases with minimal involvement of inflammatory conditions, therapeutic interventions directed at organ-specific functional impairment may prove efficacious. CRP, C-reactive protein; RBC, red blood cell; RES, reticuloendothelial system; EPO, erythropoietin. Image Credit: Authors

Based on the above results, the etiology of anemia in older patients can be broadly categorized into systemic and organ-specific factors. Systemic factors include chronic inflammation or malnutrition, both of which result in hypoalbuminemia. Additionally, age-related decline in the function of individual organ systems, such as the endocrine, renal, bone marrow, and hepatic systems, contributes to the development of anemia. In many older patients, anemia is presumed to result from the complex interplay between inflammatory pathological conditions and individual organ senescence (Figure 4). Anemia associated with inflammatory conditions partly represents a physiological response, and there are no definitive guidelines for its treatment indications [13]. However, as individual organ-specific issues are potentially manageable, it is important to actively consider the effects of aging in individual organs that coexist with chronic inflammation. Therapeutic responses to EPO or hypoxia-inducible factor-prolyl hydroxylase inhibitors are anticipated in anemia patients with limited inflammatory impact (specifically, cases exhibiting minor reductions in albumin, C-reactive protein (CRP) elevation, and modest ferritin increases).

In the present study, we defined anemia as a hemoglobin level of <11 g/dL because the study population consisted of very old adults with reduced PS. In contrast, several reports have addressed the optimal hemoglobin level in older adults, with many suggesting that higher thresholds, such as ≥13 g/dL, are more appropriate [10,14,15]. Furthermore, some studies have indicated that men may require even higher hemoglobin levels, highlighting the need to consider sex differences. Among older adults with preserved PS, the clinical significance of anemia should be regarded as greater than is generally assumed.

The limitations of this study include the following: First, this study was conducted at a single institution with a limited number of cases. Second, the patients had diverse underlying diseases, and the analyzed parameters were limited. In particular, CRP, a representative marker of inflammation, was not routinely assessed, and EPO levels were measured in only a limited number of cases. Third, eGFR may underestimate renal function when used as a method for evaluating kidney function in older patients. With these limitations in mind, identifying patients whose erythrocyte levels fall below physiologically necessary thresholds, due to decreased EPO production (frequently deviating from the decline in eGFR) and/or reduced bone marrow responsiveness, is crucial for preserving long-term functional status in older adults. Enhanced understanding of anemia pathophysiology and management strategies is expected to contribute to maintaining better quality of life in the older population.

## Conclusions

Anemia in the older adult population is primarily related to inflammation and malnutrition, with additional contributions from age-related changes in individual organs such as the kidneys and bone marrow. In the management of geriatric anemia, it is essential to consider organ-specific aging processes associated with underlying inflammatory conditions, with particular attention to impaired erythropoietin production that occurs independently of eGFR decline.
